# Levels of anti-insulin antibodies in diabetic retinopathy patients: an observational cross-sectional study

**DOI:** 10.2144/fsoa-2024-0002

**Published:** 2024-05-15

**Authors:** Bismark N Mohammed, Emmanuel K Ofori, Christian N Adekena, Seth K Amponsah, Henry Asare-Anane, Seth D Amanquah, Mubarak Abdul-Rahman, Kwesi N Amissah-Arthur

**Affiliations:** 1 Department of Chemical Pathology, University of Ghana Medical School, Accra, Ghana; 2 University of Ghana Medical Center, Accra, Ghana; 3 Department of Medical Pharmacology, University of Ghana Medical School, Accra, Ghana; 4 Department of Pathology, University of Ghana Medical School, Accra, Ghana; 5 Eye Unit, Department of Surgery, University of Ghana Medical School, Accra, Ghana

**Keywords:** autoantibodies, diabetes mellitus, glycemia, insulin, retinopathy

## Abstract

**Aim:** This study evaluated the levels of anti-insulin antibodies (AIAs) and the influence of some antidiabetic medications on AIA in diabetes mellitus (DM) patients with retinopathy. **Patient & methods:** An observational cross-sectional study. **Results:** A lower titer of AIA IgG was observed in the diabetic retinopathy (DR) and DM-only study categories compared with the control group [DR = 86 (5–560), DM-only = 50 (5–500), versus control = 200 (7–565); p = 0.017]. Taking nifedipine and metformin were negatively correlated (r = -0.32, p = 0.04) with the levels of AIA IgE in the DR group. **Conclusion:** A decreased titer of circulating AIAs was observed in the DR study category, suggesting that AIA may not contribute to the pathogenesis of DR.

## Plain language summary

Diabetic retinopathy (DR) is the main reason people lose their sight in countries with few resources. Anti-insulin antibodies, or AIAs, help the body fight off infections and may play a role in the development of DR. The study looked at how much AIA was in DR patients and how some diabetes drugs affected AIA levels. There was a negative link between nifedipine and one AIA (IgE) in people with DR, but a positive link between metformin and another AIA (IgG). AIA levels were lower in the DR study group, which suggests that AIA may not cause DR.

## Abstract (plain language summary)

Diabetic retinopathy (DR) is a significant cause of vision impairment and blindness among people in resource-limited countries.

Increasing concentrations of anti-insulin antibodies (AIAs) are seen in diabetic patients.

Whether changes in AIAs are associated with diabetic complications merits further research.

As reported by prior studies, hyperglycemia was a possible factor contributing to DR.

A decreased titer of circulating AIAs was observed in the diabetic retinopathy study category, suggesting that AIA may not contribute to the pathogenesis of DR.

Taking nifedipine was found to be negatively correlated with the levels of AIA IgE while taking metformin was positively correlated with the levels of AIA IgG in the DR group.

However, the consequences of circulating AIA in health and disease merit further research.

Retinopathy is a common microvascular complication of diabetes mellitus (DM). Diabetic retinopathy (DR) continues to cause visual impairment and blindness in several populations [^1-3^]. DR occurs as a result of metabolic changes leading to injury of the microvasculature of the retina [4,5]. The two main types of DR are the less severe form (nonproliferative) and the severe form (proliferative). Nonproliferative DR is characterized by microaneurysms, superficial or deep retinal hemorrhages, hard exudates and macular edema [6]. On the other hand, proliferative DR involves the formation of new blood vessels (angiogenesis) in the retina that may cause retinal scarring [7].

The prevalence of DM and DR is on the rise globally, and Ghana is no exception. The prevalence of DR in Africa ranges between 7.0 and 62.4% [3,8,9]. In Ghana, there is an increase in visual complications and blindness burden due to DR. The National Health Insurance Scheme spends an average of US $2500–5000 per annum on diabetes management, which is expected to be much higher for DR [10]. Screening and prompt treatment of DR appear to be disproportionate and sometimes nonexistent in many developing countries [11]. DR brings substantial economic loss to patients and their families, healthcare systems and national economies [12,13].

Anti-insulin antibodies (AIAs) are glycoproteins produced by the immune system. AIA is important in the body's defense against pathogens [14]. Their levels are determined routinely in clinical practice to provide information on the humoral immune status of patients. Although the exact mechanism remains elusive, increased circulating AIAs in DM patients have been linked to poor glycemic control and the development of insulin resistance [15,16]. AIA is suggested to impact insulin's effectiveness when it attaches to the insulin receptor, perhaps leading to a decrease in glucose uptake by the cells and resulting in hyperglycemia. Changes in AIA levels may have additional involvement in diabetic complications, leading to a further decline in productivity and potentially higher mortality rates. In DR, there is the observed upregulation of molecules associated with inflammation and increasing vascular permeability in the retina [^17-19^]. These immunoglobulins may be responsible for allergies by increasing the porosity of the blood–retinal barrier, causing leakage, pericyte and endothelial cell loss and retinal neovascularization [20]. Levels of some AIAs, which may serve as early signs in the etiogenesis of diabetic complications, have barely been studied in resource-poor settings. The present study sought to evaluate levels of AIA among patients with and without DR in Ghana.

## Materials & methods

### Study design, site, participants & clinical assessment

The study was an observational cross-sectional one. Recruitment of study participants was carried out at the National Diabetes Management and Research Center (NDMRC) and the Eye Unit of the Korle-Bu Teaching Hospital (KBTH). Prior to recruitment, participants were required to sign an informed consent form stating their willingness to participate in the study. They were informed of the following: the reason and duration of the study, the benefits of the study, the materials used in sample collection, and the potential risks of the sampling method. A standard questionnaire obtained demographic characteristics and health status of study participants. Blood was collected from each consented participant. Blood sample analysis was carried out at the Chemical Pathology Laboratory, University of Ghana Medical School. The population studied included 40 subjects with DR, 25 with DM without known complications, and 25 nondiabetic subjects (control group). For minimum sample size determination, we established that 25 persons for each study group were adequate for this study. We used a 7.0% prevalence rate for diabetic retinopathy in Africa at a 95% confidence interval and assumed a marginal error of 10%. A random sampling approach was employed, whereby an initial random sample was selected from each stratum, and then every second case was included until the desired sample size for each stratum was achieved.

All volunteers aged 25 years and above who met the criteria and who gave written consent were recruited. DR subjects were patients who had been diagnosed with the condition by an ophthalmologist specialist at the Eye Unit of the hospital. The DM group were patients without any known complications who were being managed at the NDMRC. Control subjects for the study were screened and recruited from the Eye Unit. The control group had no history of T2DM and had fasting plasma glucose (FPG) lower than 5.6 mmol/l. Individuals with familial hyperlipidemia and opaque ocular media were excluded. Patients who were immunosuppressed, such as those with immunoglobulin deficiency syndrome, human immunodeficiency virus (HIV) and hepatitis B, were excluded. Hypercholesterolemia or hypertriglyceridemia lead to lipid buildup in macrophages and other immune cells, promoting inflammatory reactions. Immunodeficiency conditions and hepatitis B infection usually result in changes in immunoglobulin levels, potentially impacting observed outcomes. Also, those who tested positive for the urine nitrite test or patients with bacterial and parasitic infections (known to affect immunoglobin levels) were excluded. All participants' body weight and height were measured using a standard physician's scale (Omron, USA) and a mounted meter rule to the nearest 1.0 kg and 0.005 m, respectively. Body mass index (BMI) was determined by dividing weight by the square of height (kg/m^2^). Participants' blood pressure was measured in a sitting position after 10–15 min of rest, using an automatic cuff blood pressure machine and stethoscope.

### Sample collection & laboratory procedures

Five milliliters (5 ml) of venous blood were taken from each participant by a certified phlebotomist. Two (2) ml of patients' venous blood was put into fluoride tubes and centrifuged at 3000 rpm for 10 min at room temperature. Afterward, FBG was determined by the glucose oxidase method by an automated chemical analyzer (Randox Daytona, UK). FBG values below 5.6 mmol/l are deemed normal [21]. The remaining 3 ml of the blood was allowed to clot and the serum was separated by centrifugation at 3000 rpm for 10 min at room temperature. Contamination by hemolysis or lipemia was avoided. The resulting sera were divided into 0.5 ml portions and placed in Eppendorf tubes. These were stored at -20 °C until analysis. After the sera were thawed (once only), the sandwich enzyme-linked immunosorbent assay (ELISA) approach (GenWay Biotech Inc. CA, USA) was used to determine the concentrations of AIA (IgE and IgG).

### Ethical considerations

Ethical approval (ID: CHS-Et/M.3-P1.9/20017-2018) was obtained from the Ethical and Protocol Review Committee (EPRC), College of Health Sciences, University of Ghana. Approval was also obtained from Korle-Bu Teaching Hospital's Scientific and Technical Committee (KBTH-STC) and Korle Bu Teaching Hospital Institutional Review Board (KBTH-IRB) (ID: KBTH-STC00040/20019).

### Data processing & statistical analysis

The data obtained were analyzed with Statistical Products and Services Solutions (SPSS), version 25. A preliminary analysis was carried out to check the quality of the data, detect potential outliers, and see if the data violated normality, linearity, homoscedasticity, or residual independence. This was checked using the Shapiro-Wilk test of normality and the assumption was observed to be violated (p < 0.05). Descriptive statistics (median, minimum, and maximum) were summarized. The Kruskal–Wallis and Tukey HSD *post-hoc* test was used to compare the clinical and biochemical parameters of the three groups. Pearson's product-moment correlation was used to determine associations between AIA IgE and IgG levels and some continuous and categorical variables. A probability value (p-value) less than 0.05 was considered statistically significant.

## Results

In total, 90 participants were recruited. Except for the control group, which had a high proportion of its participants being males (*n* = 15, 60%), the majority of the participants were females in both the DR (n = 26, 65%) and DM (n = 19, 76%) groups (Table 1). None of the participants in the control group was on any medication, whereas the participants of both the DM-only and DR groups were largely on a number of medications (Table 2). The clinical and biochemical characteristics of the study groups were also compared using the Kruskal-Wallis and *post-hoc* test analysis (Table 3). A lower titer of AIA IgG was observed in the DR and DM-only study categories compared with the control group (DR = 86 [5–560], DM-only = 50 [5–500], versus control = 200 [7–565]; p = 0.017). The DR and DM study categories were further characterized by significant elevation in FBG, BMI, age, systolic and diastolic blood pressures compared with the control group (p < 0.05). Taking nifedipine or metformin significantly influenced the levels of AIA in the DR study category. A significant negative correlation (r = -0.32, p = 0.04) was observed between AIA IgE and taking nifedipine. In contrast, a positive correlation (r = 0.32, p = 0.04) was observed between AIA IgG and taking metformin (Tables 4 & 5, respectively). Table 6 shows the multivariate logistic regression analysis used to determine the clinical and biochemical predictors in the DM-only and DR cohorts using nondiabetics as the reference category. Expectedly, FBG was found to be between twice and five-times more likely to be elevated in the DR and DM-only groups, respectively (OR: 1.96, p = 0.012; OR: 5.12, p = 0.013).

**Table 1.** Socio-demographic characteristics of the study participants.

Feature	DR group		DM only group		Control group	
	No. (40)	%	No. (25)	%	No. (25)	%
**Gender**						
Female	26	65	19	76	10	40
Male	14	35	6	24	15	60
**Education level**						
None	8	20	3	12	2	8
Basic	13	32.5	7	28	7	28
Secondary	12	30	11	44	11	44
Tertiary	7	17.5	4	16	5	20
**Occupation**						
Retired	14	35	12	48	3	12
Self-employed	22	55	13	52	17	68
Civil servant	1	2.5	0	0	0	0
Teacher	2	5	0	0	0	0
Nurse	1	2.5	0	0	2	8
Student	0	0	0	0	3	12

Data presented in frequency (No.) and percentages (%): DM: Diabetes mellitus; DR: Diabetic retinopathy.

**Table 2.** Medications used by the study participants.

Medication	DR group		DM only group	
	No. (40)	%	No. (25)	%
Metformin	38	95	23	92
Nifedipine	13	32.5	10	40
Amaryl	2	5	22	88
Lisinopril	6	15	9	36
Dionil	6	15	0	0
Insulin	5	12.5	1	4
Losartan	2	5	7	28
Atorvastatin	2	5	9	36
Amlodipine	5	12.5	8	32
Aspirin	2	5	1	4

Data was presented in frequency (No.) and percentage (%).

DM: Diabetes mellitus; DR: Diabetic retinopathy.

**Table 3.** Clinical and biochemical characteristics of study participants.

	Control (n = 25) median (min-max)	DM Only (n = 25) median (min-max)	DR (n = 40) median (min-max)	*p*-value
FBG (/mmol/l)	4.9 (3.8–6.10)^A^	7.5 (4.40–14.40)^B^	9.5 (4.40–21.40)^B^	0.001
BMI (/kg/m^2^)	23.0 (18–39)^A^	29.0 (22–42)^B^	28.0 (20–38)^B^	0.005
Age (years)	38 (23–83)^A^	65 (43–83)^B^	59 (40–76)^B^	0.001
SYS (mmHg)	134 (91–214)^A^	145 (103–203)^B^	146 (110–238)^B^	0.001
DIA (mmHg)	88 (57–114)^B^	80 (55–102)^A^	84 (60–127)^B^	0.001
IgE (IU/ml)	197.6 (6.8–477.6)	55.5 (7.9–519.2)	101.5 (7.8–763.7)	0.115
IgG (mg/ml)	200 (7–565)^A^	50 (5–500)^B^	86 (5–560)^B^	0.017

A p-value < 0.05 was considered statistically significant. A and B denote significant differences between groups (one-way ANOVA with Tukey HSD posthoc analyses).

DIA: Diastolic blood pressure; FBG: Fasting blood glucose; IgE: Immunoglobulin E; IgG: Immunoglobulin G; Max: Maximum; Min: Minimum; SYS: Systolic blood pressure.

**Table 4.** Association between participants' features and anti-insulin IgE antibodies.

Participants' features	Retinopathy group		Diabetes only group		Control group	
	r	p-value	r	p-value	r	p-value
Age	-0.04	0.83	0.01	0.97	0.30	0.15
Gender	0.22	0.18	0.21	0.31	0.07	0.74
BMI	-0.13	0.43	-0.12	0.58	0.13	0.55
Educational level	-0.11	0.51	0.13	0.53	-0.37	0.07
Systolic BP	-0.01	0.97	-0.09	0.67	0.24	0.24
Diastolic BP	0.06	0.74	-0.16	0.45	0.05	0.83
Occupation	0.05	0.78	0.01	0.98	-0.37	0.07
Fasting blood glucose	0.10	0.52	-0.30	0.15	-0.27	0.19
Metformin	-0.16	0.33	-0.20	0.92	N/A	N/A
Lisinopril	0.13	0.44	0.06	0.77	N/A	N/A
Amaryl	-0.21	0.18	0.23	0.28	N/A	N/A
Nifedipine	-0.32^†^	0.04	0.15	0.47	N/A	N/A
Losartan	-0.22	0.17	0.13	0.52	N/A	N/A
Atorvastatin	0.03	0.84	0.01	0.96	N/A	N/A
Amlodipine	-0.14	0.37	-0.07	0.73	N/A	N/A
Aspirin	0.11	0.49	-0.21	0.30	N/A	N/A

† Significant at 0.05 alpha level.

N/A: Not applicable.

**Table 5.** Association between participants' features and anti-insulin IgG antibodies.

Participants' features	Retinopathy group		Diabetes group		Control group	
	r	p-value	r	p-value	r	p-value
Age	-0.18	0.26	-0.02	0.91	0.07	0.73
Gender	0.22	0.18	0.22	0.28	0.13	0.53
BMI	-0.21	0.19	-0.14	0.51	0.03	0.88
Educational level	0.003	0.98	0.16	0.45	-0.05	0.81
Systolic BP	-0.01	0.96	-0.08	0.72	0.06	0.76
Diastolic BP	0.06	0.73	-0.14	0.49	0.01	0.98
Occupation	0.17	0.30	0.01	0.98	-0.26	0.21
Fasting blood glucose	0.04	0.79	-0.30	0.15	-0.22	0.29
Metformin	0.32^†^	0.04	-0.02	0.94	N/A	N/A
Lisinopril	0.07	0.69	0.04	0.86	N/A	N/A
Amaryl	-0.04	0.83	0.20	0.34	N/A	N/A
Nifedipine	-0.04	0.79	0.15	0.47	N/A	N/A
Losartan	-0.77	0.64	0.16	0.46	N/A	N/A
Atorvastatin	-0.10	0.55	0.02	0.93	N/A	N/A
Aspirin	-0.20	0.22	-0.21	0.31	N/A	N/A

† Significant at 0.05 alpha level.

N/A: Not applicable.

**Table 6.** Multivariate analysis to determine predictors among study participants.

Features	OR (95% CI)	p-value
**Diabetes only**		
Age (year)	1.12 (0.95–1.32)	0.17
BMI (kg/m^2^)	1.00 (0.71–1.39 )	0.97
Systolic BP (mmHg)	1.21 (1.02–1.43)	0.03
Diastolic BP(mmHg)	0.60 (0.042–0.87)	0.007
FBG (mmol/l)	5.13 (3.09–7.71)	0.013
Serum IgE (IU/ml)	0.99 (0.97–1.00)	0.139
Serum IgG (ng/ml)	1.01 (1.00–1.01)	0.081
**Diabetic retinopathy**		
Age (year)	1.04 (0.89–1.21)	0.629
BMI (kg/m^2^)	0.88 (0.63–1.23)	0.461
Systolic BP (mmHg)	1.21 (1.02–1.43)	0.028
Diastolic BP(mmHg)	0.65 (0.45–0.94)	0.021
FBG (mmol/l)	1.97 (1.12–3.44)	0.012
Serum IgE (IU/ml)	0.99 (0.98–1.01)	0.274
Serum IgG (ng/ml	1.01 (1.00–1.02)	0.090

p-value <0.05 is considered significant.

FBG: Fasting blood glucose; OR: Odd ratios.

## Discussion

The primary objective of this study was to assess circulating levels of AIA IgE and IgG across three distinct groups: adults without diabetes, individuals with DM without retinopathy, and individuals with DR. To the best of our knowledge, this research represents the first study to investigate AIA IgE and IgG levels in a cohort of participants in the sub-Saharan African. An additional objective of this study was to examine the relationship between the characteristics of participants and the levels of AIA IgE and IgG in each study group. Significant variations in the levels of AIA IgG, but not IgE, were identified across the study groups. Surprisingly, there were lower titers of AIA in the DR and DM-groups compared with the control category. The finding of lower titers of AIA in DR patients suggests that AIA may not be a contributor to the pathogenesis of DR. Indeed, the etiology of DR may be attributed to chronic metabolic dysfunction linked to hyperglycemia and insulin resistance in adult humans [22,23]. Elevated blood glucose levels have the potential to induce heightened production of certain molecules involved in the inflammatory response. Moreover, the loss of pericytes, neovascularization and accelerated endothelial proliferation in retina capillaries can potentially lead to an increase in vascular permeability [24]. This, in turn, can cause the breakdown of the blood–retina barrier (BRB), enabling the accumulation of fluids in the deep retinal layers [25]. Consequently, sustained glycemia can lead to damage of photoreceptors and other neural tissues, ultimately leading to macula edema and subsequent visual loss in individuals with DR [26,27].

Although not observed in the present study, previous studies have reported elevated levels of AIA IgE in individuals with DM. This is known to be due to allergic reactions and the occurrence of increased microvascular permeability in patients with DR [28,29]. Accordingly, it has been documented that IgE AIAs have a greater affinity for mast cells and basophil activation, producing inflammatory mediators such as histamine, leukotrienes and prostaglandins [30]. The aforementioned inflammatory mediators have the potential to enhance vascular permeability, induce endothelial cell loss, exacerbate retinal inflammation and increase oxidative stress and cellular apoptosis in the retina [31,32]. These characteristics represent the primary pathophysiological hallmarks of DR. The lack of variation in AIA IgE observed in individuals with DM and DR in this study may be attributed to the fact that these individuals were receiving prescribed drugs which could have been a confounder and influenced the observed outcome. The present study also revealed notable correlations between the administration of Nefidipine or metformin and the levels of anti-insulin IgE or IgG antibodies. AIAs affect blood glucose control especially in patients on insulin therapy [33]. Our finding however disagreed with a recent study where low AIAs were observed in DM patients using sulfonylureas and metformin [34].

The study observed that the diabetic groups exhibited significantly higher levels of BMI, SBP and elevated FBG compared with the nondiabetic control group. The aforementioned observation corroborates other studies that have reported similar patterns [35,36]. Moreover, it has been observed that there is a tendency for worsening glycemic state in individuals with a high BMI, as well as increased SBP and DBP [37,38]. Hypertension is widely believed to play a role in the advancement of DR due to the mechanical stretching and shear stress experienced by endothelial cells. The heightened viscosity of the blood associated with hypertension further contributes to endothelial dysfunction. Further to this, the endocrine system plays a role in the regulation of BP and is also separately involved in the development of DR [39].

It is noteworthy to mention that the individuals in this study who were diagnosed with DM and DR were receiving antidiabetic drugs. DM patients who developed hypertension were also prescribed anti-hypertensive drugs. These drugs may have contributed to the similarly comparable diastolic blood pressures in the diabetic cohorts. Nonetheless, the observation of notably elevated SBP readings in the case cohorts implies a potential lack of adherence to the prescribed anti-hypertensive medication.

The multivariable logistic regression analysis conducted on both the DM and DR cohorts indicated that both SBP and FBG exhibited potential predictive capabilities in these groups. Chronic hyperglycemia serves as the causative factor for many forms of microvascular illnesses in individuals with DM [40]. The aforementioned findings corroborated the assertions made by the United Kingdom Prospective Diabetes Study (UKPDS), which has primarily examined the associations between hypertension and visual impairment in individuals with diabetes. They reported the identification of an elevated relative risk associated with increased SBP with the occurrence of retinopathy [41,42]. It is widely recognized that DR can be influenced by the alterations in blood flow dynamics caused by hypertension, including compromised autoregulation and excessive perfusion. Moreover, it has been observed that hypertension, regardless of hyperglycemia, can induce an increase in the expression of vascular endothelial growth factor in retinal endothelial cells and ocular fluids. This upregulation of VEGF factor has the potential to facilitate the development of DR [43]. In addition to its effects, hyperglycemia also stimulates the enhanced activity of the sodium/glucose proximal convoluted tubule cotransporter, resulting in the retention of sodium. It is also important to consider other factors such as insulin resistance and hyperinsulinemia as potentially crucial in the etiology of hypertension, DM and DR [44].

### Limitations

The present investigation has certain constraints. The utilization of a cross-sectional design in this study imposes limitations on the extent to which possible predictors can be attributed to the observed associations. To establish causal relationships, the acquisition of follow-up data will prove to be quite beneficial. The present investigation did not differentiate between Type 2 and Type 1 nor did it have matched case-control groupings. These factors could have conceivably influenced the results of the study. The replication of this study might be conducted using bigger sample sizes, along with the utilization of an assay that effectively differentiates between anti-insulin antibodies and auto-antibodies. This study did not account for food choices and total levels of physical activity, which might be included in the design of future investigations. Additionally, the insulin-resistant state, level of glycation, and basal insulin levels of the volunteers were not taken into consideration in this investigation. Furthermore, specific sub-classes of these immunoglobulins were not measured.

## Conclusion

In conclusion, the levels of AIA IgE and IgG did not exert any discernible influence on our DM-only and DR populations, suggesting that AIAs may not contribute to the pathogenesis of DR. However, hyperglycemia and hypertension demonstrated potential predictive capabilities for the development of DM and DR, respectively. Further investigation is warranted to explore the consequences of the presence of AIAs in both the context of overall health and various disease states.
